# Seeds for effective oligonucleotide design

**DOI:** 10.1186/1471-2164-12-280

**Published:** 2011-06-01

**Authors:** Lucian Ilie, Silvana Ilie, Shima Khoshraftar, Anahita Mansouri Bigvand

**Affiliations:** 1 Department of Computer Science, University of Western Ontario, N6A 5B7, London, ON, Canada; 2 Department of Mathematics, Ryerson University, M5B 2K3, Toronto, ON, Canada

## Abstract

**Background:**

DNA oligonucleotides are a very useful tool in biology. The best algorithms for designing good DNA oligonucleotides are filtering out unsuitable regions using a seeding approach. Determining the quality of the seeds is crucial for the performance of these algorithms.

**Results:**

We present a sound framework for evaluating the quality of seeds for oligonucleotide design. The *F *- score is used to measure the accuracy of each seed. A number of natural candidates are tested: contiguous (BLAST-like), spaced, transitions-constrained, and multiple spaced seeds. Multiple spaced seeds are the best, with more seeds providing better accuracy. Single spaced and transition seeds are very close whereas, as expected, contiguous seeds come last. Increased accuracy comes at the price of reduced efficiency. An exception is that single spaced and transitions-constrained seeds are both more accurate and more efficient than contiguous ones.

**Conclusions:**

Our work confirms another application where multiple spaced seeds perform the best. It will be useful in improving the algorithms for oligonucleotide design.

## Background

An oligonucleotide is a short DNA or RNA sequence. It is usually designed to hybridize with a unique position in a target sequence. In this way the target sequence can be uniquely identified using the oligonucleotide as a probe. DNA oligonucleotides have many applications such as gene identification, PCR (polymerase chain reaction) amplification, or DNA microarrays.

Many software programs have been written to construct good DNA oligonucleotides, such as ProbeSelect [1], PROBESEL [2], ProMide [3], OligoArray [4,5], ArrayOligoSelector [6], OligoWiz [7], ROSO [8], GoArrays [9], and ProDesign [10]. One crucial issue in designing good oligonucleotides is to minimize the chance of cross-hybridization. Unsuitable regions are filtered out before checking for cross-hybridization. The underlying algorithms of these software programs are based on one or more of the following tools: suffix trees, suffix arrays, sequence alignments, seeds. Those based on seeds are very good, due to the increased accuracy and efficiency. However, their performance depends heavily on the seeds they use and our main goal here is to find the best seeds for oligonucleotide design.

Seeds were made highly popular by the sequence alignment program BLAST [11], the most widely used software program in bioinformatics. Instead of the quadratic dynamic programming exact algorithm of Smith-Waterman [12], which is infeasible for long sequences, BLAST searches for 11 contiguous matches between the sequences as an indicator of potential local similarity. It has been noticed first by [13] that considering non-consecutive matches produces better results. The first similarity search program to use this idea was PatternHunter [14] where the distribution of the match positions is also optimized. If we denote a match by a 1 and a don't care position by a *, then the default seed of BLAST is 11111111111, representing eleven consecutive matches, whereas the matches of the PatternHunter's seed are spaced: 111*1**1*1**11*111. While the distribution of the matches in this latter seed may seem random, it is not. In fact, it is optimal for this particular case, which means that any other distribution of the matches would be less effective in detecting alignments.

It is intuitively clear that several seeds, with different distribution of the matches, may detect more similarities. This idea has been used in PatternHunter II [15] where 16 seeds are used. The increase in sensitivity (that is, probability of detecting alignments) is impressive. Under similar conditions (see the Methods section), the sensitivity increases from 0.3 for the contiguous seed of BLAST to 0.467 for PatternHunter's seed and then to 0.924 for the multiple seed of Pattern-Hunter II. Multiple spaced seeds quickly became the state-of-the-art in similarity search in biological applications. They are used not only by similarity search programs such as PatternHunter [14], PatterHunter II [15], Yass [16] but also by many tools for read alignment of the next generation sequencing data, such as SHRiMP [17], PerM [18], SToRM [19]. However, there seems to be no software for oligonucleotide design that uses multiple spaced seeds. Most of them use BLAST and we found that only ProDesign [10] uses a single transition-constrained seed.

Our goal is to show that multiple spaced seeds perform the best for the task of oligonucleotide design. We shall describe a sound framework to evaluate the quality of various types of seeds for oligonucleotide search. Two aspects are to be considered: accuracy and efficiency. Accuracy is the ability of a seed to distinguish between regions that are similar with a given one and those that are not. Efficiency concerns the speed of this process.

To the best of our knowledge, there is only one study on this problem, due to Chung and Park [20]. Their conclusion is that "multiple seed selection method is not good at oligo design." This is not only incorrect but also misleading as it argues against what we believe to be the best tool for oligo design. As explained in details in the Methods section, their approach has several problems which invalidate their conclusions. Essentially, their statistical tests are incorrectly defined. In addition, they tested only some weaker variants of the multiple spaced seeds.

We introduce a different approach here and show that the multiple spaced seeds actually provide the best accuracy. The accuracy increases with the number of seeds but this comes at the price of reduced efficiency. It is interesting to notice that spaced seeds are both more accurate and more efficient than contiguous seeds.

## Methods

In this section we describe our framework for comparing various types of seeds for oligonucleotide design. We first introduce seeds and describe their working mechanism. We also introduce seed sensitivity and explain the intuitive advantages of multiple seeds.

### Seeds

A DNA sequences is seen as a string over the alphabet Σ = {*A*, *C*, *G*, *T *} whereas a seed is a string over {1, *}; a 1 stands for a match and a * for a don't care position. The number of 1's in a seed is called the *weight *of that seed and the total number of characters is its *length*. The *i*th letter of a string *s *is denoted by *s*[*i*]. A *hash *of a seed s is obtained by replacing all 1's in *s *by letters from Σ. If the weight of s is *w*, then 4*^w ^*different hashes can be obtained from *s*. A given hash of *s*, say *h*, occurs at position *i *in a DNA sequence *D *if aligning *h *with *D *starting at position *i *causes all letters of *h *to match the corresponding letters of *D*.

**Figure 1:**
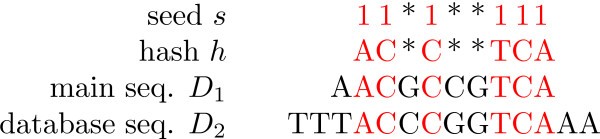
**Hit example**. An example of a hit: *s *hits *D*_2 _at position 4 as the hash *h *of *s *occurs in *D*_1 _at position 2 and in *D*_2 _at position 4.

An example of a hit is shown in Figure 1. Assume we have a DNA sequence *D*_1 _and we search for sequences similar with *D*_1 _in a database, using the seed *s*. A *hit *of *s *in the database is a position *j *in a sequence *D*_2 _in the database such that there is a hash of *s *that occurs both (at some position) in *D*_1 _and at *j *in *D*_2_.

A hit means there is a chance for an actual similarity. The ability of a seed to detect similarities is called *sensitivity*. A Bernoulli model of sequence alignments has been introduced in [15] in order to formally define sensitivity. An alignment is represented as a binary sequence, *R*, where a 1 represents a match and a 0 a mismatch between the two sequences. The probability *p *of a 1 is called *similarity*. A seed s hits *R *at a positions *i *if aligning *s *with *R *starting at *i *causes all 1's in *s *to match 1's in *R*. The sensitivity of s is formally defined as the probability that it hits *R*. It depends on both the length *N *of the random region *R *and similarity level *p*. The sensitivity of the contiguous BLAST seed for *N *= 64 and p = .7 is 0.3 whereas the sensitivity of the spaced PatternHunter seed, under the same conditions, is considerably higher: 0.467.

### Multiple spaced seeds

Multiple spaced seeds are sets of seeds. A multiple spaced seed containing *k *≥ 1 seeds will be called a *k*-*seed*. The definition of a hit is naturally extended to multiple seeds: a multiple seed hits when one of its seeds does so. The sensitivity is therefore defined similarly. A dynamic programming algorithm for computing sensitivity for multiple seeds is given in [15]. Under the same conditions N = 64, *p *= 0.7, the multiple spaced seed of PatternHunter II, consisting of 16 seeds of weight 11, has sensitivity 0.924, much higher than a single spaced seed. It is therefore natural to consider multiple spaced seeds as the best candidate to oligonucleotide design.

The sensitivity alone is not sufficient to assess the quality of a seed. That is because we can increase the sensitivity as much as we like simply by decreasing the weight. However, that would cause an increase in the number of random hits. We have therefore a trade off: decreasing the weight increases the sensitivity but also the number of random hits whereas increasing the weight decreases both. Weight 11 achieves a good balance and this is why it is used in the above mentioned programs.

More precisely, consider a single seed *s *of length *ℓ *and weight *w*. Consider also a random region *R *of length *N *and similarity level *p*, as done in the definition of sensitivity. The expected number of hits s has in *R *is (*N *- *ℓ *+ 1)*p^w^*, since there are *N *- *ℓ *+ 1 places where *s *can hit and each has probability *p^w^*. If we increase the weight of the seed by 1, then the expected number of hits becomes essentially a fraction *p *of the old one. Assuming that the four bases A, C, G, T appear with equal probability, that means that the number of expected hits is reduced to one quarter of the previous one. Less hits means less wasted ones, that is, less false positives and therefore increased specificity. However, increasing the weight of a seed also decreases the true positives, and therefore the sensitivity. In order to increase both, we can increase not only the weight but also the number of seeds. It turns out, as noticed by [15], that simultaneously increasing the weight by one and doubling the number of seeds provides slightly better sensitivity. But doubling the number of seeds only increases the expected number of hits by a factor of two whereas increasing the weight by one reduces it to a quarter. Essentially, this is the main reason why multiple spaced seeds are so good.

**Figure 2:**
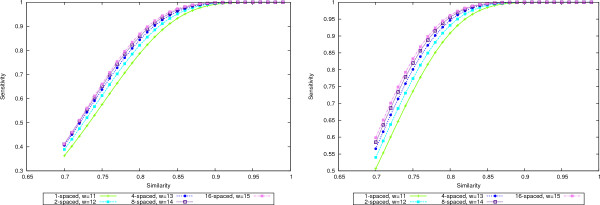
**Seed sensitivity**. Sensitivity curves for multiple spaced seeds with 1, 2, 4, 8, and 16 seeds of increasing weights: 11, 12, 13, 14, and 15, respectively. The length of the random region is *N *= 50 in the left plot and *N *= 70 in the right plot. In both cases, doubling the number of seeds outweighs the increase by one in the weight and therefore the sensitivity increases.

As an example, in Figure 2 we plot the sensitivity values for multiple spaced seeds with 1, 2, 4, 8, and 16 seeds and weight 11, 12, 13, 14, and 15, respectively. Various levels of similarity are used to give a complete picture. As it can be seen from the graphics, the sensitivities increase with the size of the seed set, even if the weights increase as well.

One should be aware however, that more memory is required for a higher number of seeds in order to store more hash tables and this enforces an upper bound on the number of seeds that can be used.

### Accuracy and Efficiency

The oligo design problem requires the ability to construct oligos that will hybridize only at unique positions in a given sequence. That is, for a given sequence (a potential oligo), we need to be able to accurately distinguish sequences that are similar with it from those that are not. Our setup will therefore include precisely constructed sequences of both types which need to be distinguished.

**Figure 3:**
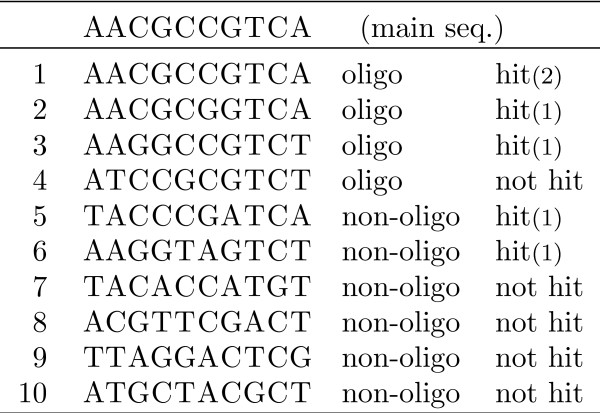
**Data set example**. One main sequence and its associated secondary sequences, marked as oligos or non-oligos. The last column shows which of the secondary sequences are hit by the seed 11*1**111 and the number in parentheses gives the number of hits.

Assume we have a set of sequences, which are divided, as in [20], into groups, each group having a main sequence and a number of secondary ones. A small example (unrealistic) of a single group is shown in Figure 3. The first sequence is the main one and the other ones are secondary. The secondary ones are divided into those sequences that are considered similar with the main, called *oligos*, and those that are not similar, *non*-*oligos*. We do not consider at this point the criteria used to distinguish those, we just assume they are given.

We define next a measure of the quality of a given seed. A widely used measure for the accuracy of a test is the *F *-score, which is the harmonic mean of precision and recall. We define the usual statistical measures of true/false positives/negatives, as follows (the hits refer to the given seed):

- *TP *is the number of oligos that are hit,

- *FP *is the number of non-oligos that are hit,

- *TN *is the number of non-oligos that are not hit,

- *FN *is the number of oligos that are not hit.

The precision *P *and the recall *R *are defined by ,  respectively, and the

*F*-score is their harmonic mean:

We shall define the *accuracy *of a seed as its *F *-score.

Note that, in binary classification, "recall" is called also "sensitivity." To avoid any confusion, we use the term "sensitivity" only with the meaning of "seed sensitivity" as defined in the "Seeds" subsection above.

In our example in Figure 3 for the seed *s *= 11*1**111, it is easy to verify that the oligos 1, 2, and 3 and the non-oligos 5 and 6 are hit. The oligo 4 and the non-oligos 7 to 10 are not hit. We have *TP *= 3, *FP *= 2, *FN *= 1, and *TN *= 4. Therefore, *P *= 0.6, *R *= 0.75 and *F *= 0.667.

The approach in [20] uses also the *F *-score as measure of accuracy (called discriminability), however, different definitions for true positives are used for precision and recall, making the *F *-score irrelevant as it involves the precision and recall of different tests.

The *efficiency*, *E*, of a seed is the reverse of the average number of seed hashes needed to detect one oligo. That means, we divide *TP *to the number of all hits in all secondary sequences:

In our example in Figure 3 the oligo 1 is hit twice, whereas the other hit sequences are hit only once each. The total number of hits is 6, *TP *= 3 and therefore the efficiency is *E *= 0.5.

The efficiency of [20] is defined by an arbitrary normalization of the average number of hits per oligo, where the average considers, incorrectly, all oligos, instead of those that are hit (*TP*). Efficient discriminability, the most significant measure of seed quality in [20], is obtained by multiplying discriminability with efficiency. This measure depends completely on the way normalization of efficiency is done and therefore impossible to interpret. In fact, by changing the normalization, most any seed can be made to appear the best. We shall not mix accuracy and efficiency.

## Results and Discussion

We compare in this section various types of seeds using the framework constructed above and then discuss the obtained results.

### Data sets

Data sets were built using the OligoGenerator program of [20]. OligoGenerator takes as input the number of target (main) sequences, the number of variations for each sequence (secondary sequences), and the length of each sequence. We have generated six data sets, three of 50-mer and three of 70-mer oligos. The number of main and secondary sequences are the same as in the data set of [20]. The criteria used were slightly different as we followed the model of He et al. [21]. For the 50-mer data set we used the following thresholds:

- identity level with target sequence: 85%

- maximum stretch of continuous matches:15bp

- hybridization free energy: -30 kcal/mol

The difference between our data set and the one of [20] is that the latter was built using -40 kcal/mol. For the 70-mer data set we used, again from [21],

- identity level with target sequence: 85%

- maximum stretch of continuous matches: 20 bp

- hybridization free energy: -40 kcal/mol

### Seeds

Computing optimal spaced seeds is a hard problem; see [15,22]. For a single seed it is feasible to try all seeds and compute the sensitivity of each to determine the optimal. Therefore, the single seeds we consider, contiguous, transition-constrained, and spaced, are the same as those of [20]. Transition-constrained seeds were introduced by [16] and used in their YASS software program. Such a seed contains, in addition to matches and don't cares, a new character, @, which stands for either a match or a transition, that is, a substitution A↔G or C↔T. The biological motivation for this is that transitions are more common than transversions, that is, A/G↔C/T. The seed used in YASS is 1@1**11**1*11@1.

Computing optimal multiple spaced seeds is significantly harder than single seeds. Even computing an optimal 2-seed of usable weight and length is infeasible. Therefore, many heuristic algorithms have been designed to compute multiple spaced seeds but they are all exponential, with the exception of SpEED [23](http://www.csd.uwo.ca/~ilie/SpEED), which is based on the notion of overlap complexity of [24]. Chung and Park used two weaker versions of multiple spaced seeds, namely BLAT and vector seeds.

Using SpEED, we have computed highly sensitive multiple spaced seeds with 2, 4, 8, and 16 seeds. The parameters used by SpEED for computing the seeds are derived from those of the oligos. That is, *N *= 50 for 50-mer oligos and *N *= 70 for 70-mer oligos. In both cases, *p *= 0.85, which is the identity level. (All seeds that we have used are given in the additional file 1.)

### Comparison

For each of the two cases, 50-mers and 70-mers, we have computed the average accuracy and efficiency for all seeds on the data sets generated. The highest accuracy values for each seed type are shown in Table 1. Both the mean and standard deviation are given, as well as the weight for which the maximum accuracy is achieved. The ranking, in increasing order of accuracy, is the same for both 50-mer and 70-mer cases: contiguous, transition, 1-seed, 2-seeds, 4-seed, 8-seed, and 16-seed. In terms of standard deviation, the results are significant, especially for the 70-mer case. The difference between the transition seeds and 1-seeds is not very large, as expected. Also, the differences between the accuracy of multiple spaced seeds essentially decrease when the number of seeds increases.

**Table 1:** Highest accuracy values

**50-mer data sets**				**70-mer data sets**			
	
**seed type**	**max. accuracy**		**weight**	**seed type**	**max. accuracy**		**weight**
					
	**mean**	**stdev.**			**mean**	**stdev.**	
	
contiguous	0.8760	0.0011	10	contiguous	0.8822	0.0003	11
transition	0.8856	0.0013	12	transition	0.8985	0.0002	13
1-seed	0.8888	0.0017	12	1-seed	0.9009	0.0001	13
2-seed	0.9018	0.0019	13	2-seed	0.9082	0.0006	14
4-seed	0.9051	0.0014	15	4-seed	0.9138	0.0003	16
8-seed	0.9080	0.0018	16	8-seed	0.9176	0.0008	17
16-seed	0.9117	0.0013	17	16-seed	0.9191	0.0006	19

The highest accuracy for each seed type is given; for the 50-mer data set in the left table and the 70-mer data set in the right table. Both the mean and standard deviation are given. The weight of the seed that achieves the highest accuracy in a given category is given in the last column.

**Figure 4:**
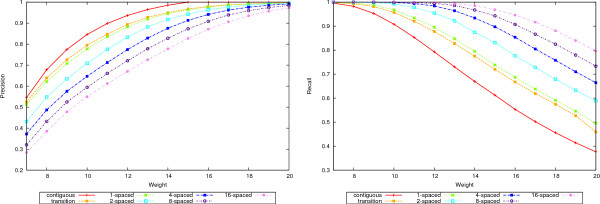
**Precision and recall for 50-mer data sets**. The left plot shows the precision and the right plot the recall values for the 50-mer data sets.

**Figure 5:**
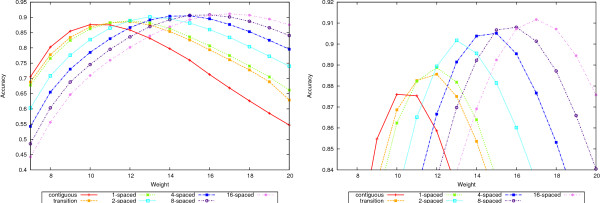
**Accuracy for 50-mer data sets**. The left plot shows the accuracy values for the 50-mer data sets. The right plot shows the top part of the curves to emphasize the differences.

**Figure 6:**
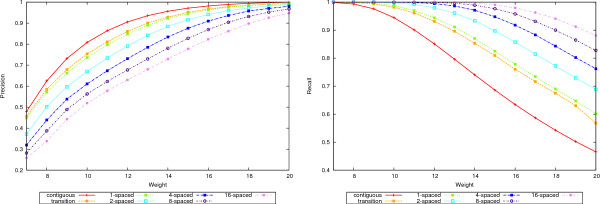
**Precision and recall for 70-mer data sets**. The left plot shows the precision and the right plot the recall values for the 70-mer data sets.

**Figure 7:**
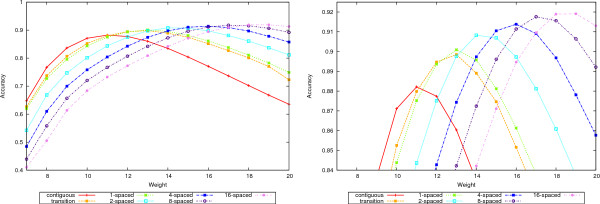
**Accuracy for 70-mer data sets**. The left plot shows the accuracy values for the 70-mer data sets. The right plot shows the top part of the curves to emphasize the differences.

A complete picture is given in Figures 4, 5, 6, and 7 where the precision, recall and accuracy are plotted for all seed types, for weights between 7 and 20. As expected, precision (left plots in Figures 4 and 6) increases with weight whereas recall (right plots in Figures 4 and 6) decreases. For high weight the precision approaches 1 for all seeds and so does the recall for low weights. Therefore, the accuracy will have a maximum in-between. Figures 5 and 7 give the accuracy values, with the complete curves in the left plots and the top part of the curves enlarged in the right plots. It can be seen that more seeds will have a higher maximum, which is achieved for higher weights. (The complete results for our tests for all 50-mer and 70-mer data sets are given in the additional files 2 and 3, respectively.)

**Figure 8:**
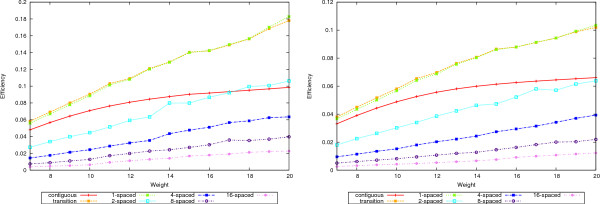
**Efficiency**. The efficiency values for the 50-mer data sets are shown in the left plot and for 70-mers in the right plot.

The increased accuracy comes at a price in efficiency. Figure 8 shows the efficiency curves for 50-mers in the left plot and 70-mers in the right. Increasing the number of seeds decreases the efficiency. Notice however that both 1-seeds and transition seeds are more efficient than the contiguous ones. Between the former two, the 1-seeds are slightly more efficient than the transition ones.

**Figure 9:**
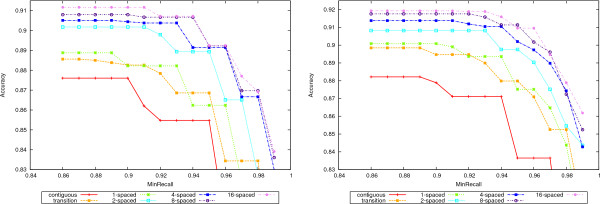
**Accuracy for bounded recall**. The accuracy values for bounded recall values are given for the 50-mer data sets in the left plot and 70-mers in the right one. For each value × on the abscissa, only the accuracy values of seeds with recall at least *x *are considered.

**Figure 10:**
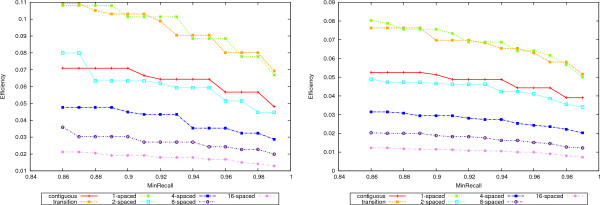
**Efficiency for bounded recall**. The efficiency values for bounded recall values are given for the 50-mer data sets in the left plot and 70-mers in the right one. For each value *x *on the abscissa, only the efficiency values of seeds with recall at least *x *are considered.

In the process of designing oligonucleotides, similar regions need to be identified and eliminated in order to keep the unique ones, out of which oligos can be chosen. For this purpose, a very high recall is desired. Therefore, we shall also rank the seeds by setting a lower bound on the recall and then considering only the accuracy of those seeds that satisfy this lower bound. The values for the bounds on the recall values are 0:86, 0.87,..., 0.99. Figure 9 shows again the superior accuracy of multiple spaced seeds, as seen from this perspective. The same ranking of the seeds as above is observed. Finally, Figure 10 gives the corresponding efficiency values for the recall lower bounds.

A last comment concerns the transition seeds. A single transition seed is slightly less accurate than a single spaced seed. However, this reason is not sufficient to rule out multiple transition seeds. Our analysis focuses on multiple spaced seeds since we were in position to compute very good ones. Multiple transition seeds should be investigated further.

## Discussion

As explained earlier, accuracy and efficiency cannot be mixed. Taken separately, they show clearly the ranking. Together, they give the trade off: better accuracy comes with a price in efficiency (except when contiguous seeds are replaced by transition-constrained or single spaced seeds).

## Conclusions

We have presented a sound framework to compare seeds for oligonucleotide design. It is known that multiple spaced seeds perform better than the other seeds in many applications but the requirements of oligo design are different. We have proved that, also in this application, multiple spaced seeds have the highest accuracy. This corrects the conclusion of Chung and Park [20]. We hope that our study will determine researchers in this area to use multiple spaced seeds in software programs for oligonucleotide design. The seeds can be created using the SpEED program that we mentioned before. The assessment of the seeds can be done using a framework as above.

## Competing interests

The authors declare that they have no competing interests.

## Authors' contributions

LI and SI identified the error in the approach of [20], proposed multiple spaced seeds as the best candidate, and designed the new framework. SK implemented the approach, built the data sets using the programs of [20], and compared the seeds. AMB used SpEED to construct the multiple spaced seeds. All authors read and approved the final version of the manuscript.

## Supplementary Material

Additional file 1 **This file contains all the seeds used in our tests**. The contiguous, transition, and single spaced seeds are the same as in [20]. The multiple spaced seeds were computed using SpEED. Click here for file

Additional file 2 **This file contains the complete results of our tests for all the 50-mer data sets**. Click here for file

Additional file 3 **This file contains the complete results of our tests for all the 70-mer data sets**. Click here for file
