# Supplementary material for: Seeds for effective oligonucleotide design
Source: BMC Genomics. 2011 Jun 1;12:280. doi: 10.1186/1471-2164-12-280 (PMC3128067; doi:10.1186/1471-2164-12-280)
Supplement: Additional file 1 — This file contains all the seeds used in our tests. The contiguous, transition, and single spaced seeds are the same as in [20]. The multiple spaced seeds were computed using SpEED. [file 1471-2164-12-280-S1.PDF]

N=50, k=2

w=7

1101010111
11100001001011

w=8

111001101011
11101000010001011

w=9

1110110100111
111010000100010111

w=10

1111011010111
111010001001001111

w=11

111010110110111
1111010010001010111

w=12

1110101101101111
1111010001001000110111

w=13

1111011011101111
1111011000010100110111

w=14

1111011001101010111
11110101000001000010011001111

w=15

1111011001110101111
111011010000110010010101111

w=16

11101101101110101111
11101101100001010010001110111

w=17

111110110111001101011111
11111100010100100110010101111

w=18

111101110011011010111111
1111101010001001101000110011111

w=19

111101101110101111011111
1111101010011010001100011011111

w=20

111110111011110110111111
111110110100011000101001101011111

N=50, k=4

---

w=7

1101010111
1110001001011
1101001000100011
110010001000001011

w=8

11011001111
11100010101011
11010010000100111
110101000000100001011

w=9

110110101111
1110100010010111
11101001000010001011
11100010001000010001011

w=10

111001011010111
110111000111011
1110101000100100111
1110100100000000100010111

w=11

111011011010111
11101001010001001111
11010100011100011011
1111001000000100100010111

w=12

111101101110111
1111000101011001111
11101001001000100101111
11101001000010100010100111

w=13

1111011101101111
11110100101011001111
1110101001100001110111
11110010100010001000101111

w=14

11110110111011111
111011100010100100101111
1111000101100010001000101111
1110101001001000100100010100111

w=15

111111011011101111
1110111000011010010110111
11110010010100100010100011111
11110001010001000100001100101111

w=16

11111101110101101111
11110101100110010001011111
11101100100001010100111001111
11110100001100001001001001110111

w=17

111110111011101101111
1111010010110010100011101111
1111010111000000110010010110111
1111100100001100010010101000101111

w=18

1111110111101011101111
1110110100110101001100111111
1110111010000111001001011100111
1111100101001100000100010100111111

w=19

11110111011101111011111
11111101101001110001010110111
11110101011001000101100011011111
11110110001000101101000101100101111

w=20

11111101111101111011111
11110110011101000110110111111
111101110100100110110001010111111
1111110010101101001000011001010101111

N=50, k=8

w=7

110110111
1110001001011
101010100000111
1110010000100011
11010000001010011
11001001000100011
11000010010001011
10100101000001011

w=8

1110110111
11100010010111
1100101000101011
10110010010001011
110100001001000111
110001010000100111
110101000010010011
111001000100001101

w=9

11110110111
1011000101001111
1101001000010101011
11010100010100100011
111100001000100010101
110010010000010100111
110001100100000011011
110010001010001001011

w=10

111011101111
11110001010010111
11101000100011000111
110101001001000101011
1101100100010100001101
1101100011000010010011
1101011000001100100101
1110000100100001010111

w=11

1111011101111
111101010000011001011
1010101100001001000010111
111001000100100010100100101
1110100001010000000100110011
110010010000010100000010010111
10100110000100010010000001111
11010000110001000010001010011

w=12

11111011101111
1110010110000100110111
111100010010001000001101011
1101000110100000101001100011
11010010100010000100010011101
101011000100010100010000011011
111100100001000010100001010011
111001000000101000100100001111

w=13

111101110111111
11101011000100100101111
111100001010001011000010111
11011010010000100001010100111
111010001000011000101001010011
1101010001000100101000001001111
1110100100010000100100011001011
1100110000101000100000110110011

w=14

1111101111011111
11101101010100001101111
111100001100010010101011011
11101100100010011000010010111
111100010011000011000010101011
110011100010010000100110101011
111010010000101000110100010111
111010010010010100001001100111

w=15

11111011110111111
111011100100100101011111
1111010010001000110100110111
111010010001001100011001001111
1111001001010001010001000111011
1110101000101100000100111000111
1110100111000010011000101001011
1100111000010100101010000101111

w=16

11111011101101011111
1111010100111000101100111 1
11111010010001101000010110111
110110011100000100110011100101 1
1110100101100100010010100011 0111
11011010000110100010100010011111
1110101100100000101001010110011 1
1110011001010110000100010101011 1

w=17

1111011101110110111 11
111101101000110010101010111 1
111100100110000101001011010101 11
11010100111000000110011000101101 11
1101101100001010100100001100011011 1
11100111001001001000011001001010111
1110101000011001101000000111100101 1
1111100101000110000010101000100111 1

w=18

11111110111110111111
11111010011001101001110111 1
111011101010010001010110001111 1
1111100001011011000001101010011 11
1110101110010001100010010010110111
110110100011100001001011100101011 1
110110101100001010110000100110111 1
1111001100010100011101000110010111

w=19

111111101111101110111 1
111101100111010010101011111 1
111011101001110001001100101111 1
111010100101101010100011100011111
11110110001000101101110000101101 11
111110101001100101100001010110011 1
1111000111100001001001011010111011
1101100110101100000011100110101111

w=20

111111101111101110111111 1
1111010101101100110011110111 1
1111011110001010010111001011011 1
111101110001000010110011101100111 1
1110110100101011100001101001011011 1
11111000110010111010000011101010111
11110011011000010111010100001110111
11101100101111000010100100110101111

N=50, k=16

w=7

110101111
11100000110101
10110010000100101
11100001001000101
110010100000010011
110001000001001101
110100000100010011
101010000001100011
1100010010000000111
1001001000100001011
1100100010000011001
1100100001100000101
1010000010100100011
1101001000001010001
1010000100001010011
1010000100010001101

w=8

1110110111
111010001000111
101001100010001011
101010000001100111
1100100100010010101
1011000001001010011
1110010000010101001
1101000010010000111
11000100001101000101
10110000100100100011
11000100010000101011
11001001000000110101
11010000110000010011
10010100001010001011
10100011000010010011
11000010101000001011

w=9

1111011111
110110100001111
111000100101001011
110101000110000111
1011100000100110011
1110001010000101011
1101100010100010101
1110010000110010101
11001001001010001101
11001010010000100111
10101001000100011011
11010011000010001011
11100100100010100011
11010000110000010111
10101000010011010011
11000110010001001011

w=10

111101101111
11011100100001111
1101100100011001011
1111000010011010101
11100010110000011011
10110011000010100111
11101000001100111001
11000110001010010111
111001010000011100011
110101001010001001011
111000101010010001101
111010001001001001011
110010100100010101011
101011100001100010011
101101010001000100111
110100100101000010111

w=11

1111011101111
110101100001000101111
1110010001000011010010101
10100110100000001100100100011
101011000000001100101001001
11100000110010000001000110011
10100101010001000100000001111
11001010000100010100000010111
110010010100010000101000011101
110001001001000100001000110111
111000010100001000010100010111
100101000011000000100110000111
110110000100100001000100100111
110110010000010010000001100011
101110000000110000010000101101
111000100001011000000001010101

w=12

111110101101111
1110011000001010011011 1
11110000100100011000010011 1
10101001101000000100011001011
110101100000011001000100001101
111001000010010000110000011011
110011000010100010000101000111
110001011001001000001000010111
1101001010100000100100100000111
1101100001000101000000010100111
1101010001001000000110001010101
1101000100010101001000001001011
1110100100000000010101001010011
1101000100101000010001100100011
1100010101000010100100010001011
1011010000011000000100110100011

w=13

111111011101111
11011100100100001101011 1
11010011010000000110001010111
110100101100000000101110000111
1010110000010010001100001010111
1110010000101010001001000101101
1110101000010100011001000100011
1111000100000101001010010010011
11010000101011000000001010011 0011
111000110001000101001000000011011
111010001010000100010000011001011
101001100100001100000101001001 11
110010100001001010001001000011 11
110110000110000011000010001001 11
101101100000011001000000011010101
110001010100010001010001010010 11

w=14

1111101111011111
11011110001010001001111 1
11101100010010100011000101 11
110101001100001010010010010111
1011001011000000001110101000111
1010110000110100000001110100111
1110000110100100100010001100111
1110101000001010011001001001011
11100101001000110000100100010111
11101001000110001010000100101011
10111010001001000110000001101101
11000110010101000010100001110011
11011001000010011000101010000111
11100100100010100100101000101011
11011000101000100010000110011011
11110000011001001001010010100011

w=15

111110110111101111
111011100011000100100111111
111011001000100010101101001011
110011100100011001001000010110 11
110110100100001010100100110000111
110101001101000000110001010110011
111010001001001100001001101000111
101101000110100000101010011001011
1110100100000010011001100000101 0111
101001110000101000010101000100 1111
101110001011000100100100000011 00111
111001001001011000011000010011 0011
111100010100010100010001100010 1011
110101100001001011000000101001 0111
110010101010000101000011000011 1011
110100010011100000000110101001 1011

w=16

11111110111101111 1
111011110001010010011101 11
111100101100000010101110100111
111100010011001010000100110110 11
110110001010100011011000000111011
111110010100100000110010101000111
110101100010001010010011001001111
110011010010001101000100101001111
111010000110011010100010001010 0111
110101010101000100000111000011 0111
110011010000101011000000101011 01011
111100101000000110001000111001 01011
110100110010010001100100000111 01011
110101001011100000101000101100 1011
111000110010100100101100010001 0111
110101011000011000101010010001 1101

w=17

111111110111101111 1
1111001110010011001101011 11
11111100100001100010011101010 11
111010011001000111100001001011 11
111001010101010110001010000110111
110101100100110000010100011111011
111010111010000010100110010110011
111100000111001010100001011100111
111100010110010000010101100101 1011
110110001100101001101010001100 1011
111010101001010001001101001100 0111
110110011000110100100000111001 00111
111001100011000000111010001001 11011
111010010100101001011001010001 0111
111011000001101010000110100001 1111
110101101001001100101000010101 0111

w=18

1111110111011110111111
1111011010011101000101011111
11101010101101100000101001101111
111101000111000100011010010110111
1100111001001000101100100111010111
1111010010101000110011000101011011
1110010110000100101100011100110111
1110111000100010110001101000111011
11011100101100000111000100110100111
11011101000001111001000010110011011
11001011001101010000110011010100111
11101001100001110101011000000101111
11101000011011000100100111000011111
11110011010000100000101111001100011 1
11110100110010000101010100101011011
11100101011010010110000010011101011

w=19

11111011011111011101111
11110110011011000110101011111
11110111001101010001001011010111
111010101100001110110110001001111
1110110010010111100010101100100111
1101101100100000111110000011001110 11
1101010111001010001100100111100111
1101100011111001010010001010011111
11101011010001100110101100000011111
11110100101010110000101100001111011
11101100111000000101011011001011011
11110011010100001100011001011101011
11011111000010010100100111011010101 1
11110010100111100010000011010110111
11010101100101000001110101110010111
11110101010001001011100010101100111

w=20

111111111011111111011111
11110111100011100101010111111
11101110010011101101001001011111
111001011111001001000111100110111
1101110011100010001101011011010111
1110110100001101000111110010110111
1011111000101011001011001110001111
1110110011010111100000010110101111
11100101011000111011001101000111011
11011100001101110101010100001110111
11011011010010011011100100011100111
11101010110110000110000110111011011
11101101100101010100100011010101111
11111001101000010010111000111011011
11110011010101001100111010001001111
11101010110110000101011001100101111

N=70, k=2

w=7

1101010111
11100001001011

w=8

111001010111
11010100000010010011

w=9

1110110010111
11010010100000001000111

w=10

111010010110111
110100100010001010001111

w=11

1110110101001111
1101001100100000001010111

w=12

11101001010110011111
1111010001000101000011011

w=13

111101011001101111
11101010010010000000110100111

w=14

11110110011010101111
111101010000010000100110011111

w=15

11110110011101011111
111011010000110010010101111

w=16

1111001110110101011111
11111001010000011000100101101111

w=17

1111100110011010101101111
11110100101000100011000001011001111

w=18

111110011011010101011101111
11101110010010100011010010011111

w=19

11111011100101110101101111
111011011010010000101010000110011111

w=20

111110101110110110011101111
111011100100010100100001010110011111

N=70, k=4

w=7

11101010011
101001001000111
11001000010100011
110100010000001011

w=8

1110110111
1101010001001101
1100101000010001011
1100010000010010000111

w=9

1110110100111
111000100101001011
110010100000100010111
11100100010000010010011

w=10

11101101010111
1111001001000100111
110101000001011001011
11010001100001000010111

w=11

1111001101010111
1110101000011000011011
1101001001000100010001111
110110001000000010100101011

w=12

11110110011010111
1110100010010010100111 1
111010100100001001000101111
111001000010100000010001011111

w=13

1111011101101111
11110110001001001010111
111010010100000110100011011
1110100000101010001000011001111

w=14

11110110111011111
11101110001010010010111 1
111100010110001000100010111 1
11101010010010001001000101001111

w=15

1111011101011011111
111010101100010010001101111
11101000110010100001001001101111
1111011000100000100100001100010111

w=16

111101110111011011111
11101111000010100100110101111
111101001000101010001001100011111
1111001001100001000010110001001011 1

w=17

1111101101011101111111
111011101000110010001011011111
1111001100010100100001101010011111
111101000100100101000010001001100111 1

w=18

11110111011110110111111
1111010110010101001000110011111 1
11101100010110000011000101000110111 1
111100101100010001000010011000001101011 1

w=19

1111100111001101010110111111
1111010100110100100110001100101111
111101011000000101100100010111010011 1
11111001010011001000010100001000101011 11

w=20

1111100111010110101110111111
11110110010001010111000001110110111
111101010110010100001001100010001101111
1111101001010001000010010000011100001101111

N=70, k=8

---

w=7

111101011
110100000100111
110000010001001101
1001000100000001010011
10101000010000100011
101000010001001000011
110010000000100001011
110000010010000010101

w=8

1110101111
11001100000101101
1101000000100010001111
11000100001000010010011
101010000001000000010011001
11000100010010000000001011
11010000100100000010000011
11010000001000100100000101

w=9

11101101111
1101100000010100111
1100100001010000010111
1101010000010000010001011
11001000100001001010001001
11000010010001000000110011
11010000101000100000001011
11100001000100001001000011

w=10

111101101111
110100100010110011
111010001000010010100111
11101000010010010001000011
11000101000100000101000001111
101100000010010000010001011
1110001000100010000000110101
11001001000001010000000101011

w=11

1111011101111
11001101000010010111
1
11010010001000101010000111
11011000010100001000101001 1
1110000101001000000100110011
1100110010000100010000101101
1110001000010010010010001011
1010100010000001100100001 0111

w=12

11111011101111
1110010110000100110111
11110001001000100001101011
110100011010000010100110001 1
11010010100010000100010011101
101011000100010100010000011011
111100100001000010100001010011
111001000000101000100100001111

w=13

111101110111111
11101011000100100101111
111100001010001011000010111
1101101001000010000101010 0111
1110100010000110001010010100 11
11010100010001001010000001001111
11101001000100001001000110010 11
11001100001010001000000110110011

w=14

1111011101101 1111
1110110001010100110 1111
111101010010010001011 00111
1101100011000010010101010111
11010101001100000010110001111
11100110100010001100100001111
11010011000011100001000110111
11110010010100010100001011011

w=15

1111101111011 1111
11111010001001010011 10111
1101100101010010011010 0010111
11010011000110001010001001 01111
1110010100100110001000100110 1011
11111000010100000100001101001 1011
111001100000110100001101000010111
111010101100000001010100001100 0111

w=16

1111011101111011111
11101101100011010000110111 1
110110101100000100101011000111 1
1101110000011100010001010001110 11
1111010010100010001100000101100111
11110001100100010100000110010010111
11100010011010001001001001010010111
11011001100001000010101001000101111

w=17

111110111101110111 11
11011011100010100100110101111
111011100010001001001000110010 1111
111010100101001000100110000100111011
1100110100011100000010101000011010111
11011010000001010110010000011000110111
11110100011000011000010010010001011011
11110001100100000101000010101001001111

w=18

11111011110111011011 11
1101110110000101011000001110111 1
11111000100101001010001010010011011 1
11010101100100000100011000010110010111 1
1110101100101100000000101100010011000111
11011100001010001010001001100010100101 11
111100001000100110010010000001101010011 11
111001101010000111000000011000000101110 11

w=19

1111011110110111011111 11
11101110100100011100100101 11111
111110100100100010101001110001011 11
1111001010100011001000001100011101011 1
111011000011011000010000101101011010 11
1110101100000110010011010100000110011 11
1111010011100001000011010001010100011 11
1111000100110100101010000011001001101 11

w=20

11111011101101111011111 11
11101111000101101100000101011101 11
111101101001100010001010101101000111 1
1110111000101010000110011000010110101 11
11111000101000110100100001101000100111 11
111101000110001001100000110100010011100111
111001011010010010010101000011100001110 11
111100100101110000001010000101001101001111

N=70, k=16

w=7

11110111
110100000110101
1110000100010000101
10010100010100000011
110000010100000100011
110001001000000010101
101100000010001000011
101001000100001001001
1100001000000100010011
1010000010001001000011
1010001000010000000111
1000100100100000100011
1001000100000100010101
1100100000011000001001
1010010000100000011001
1011000000000100101001

w=8

1111010111
11101000000001001101
111000001010000000100101
10010001000010010000111
10100010001000010010011
110010010000001000100011
110000100100000010100101
110000101001000000011001
101001000010000010001011
1100001010000100001000101
1100010000001010000001101
1000110000100000001011001
1001000100010010000110001
1100100000100000010100011
1101000000001100000010011
1010100001000001000001011

w=9

11110110111
1111000100000110101
1100010100000000011001011
110010001001000010000011 1
11000110000100010000001011
10110100000001000100001101
11001000001001000100100011
10100010010000100101000011
101001010010000001000100011
110001010000001000001000111
111000000101010000001001001
110100000100100000010100011
101010100010000100000001011
100011001000010010000010011
111000001000100100100000101
100110000100000011000010101

w=10

1111010110111
11010010010010001111
10110100001000110000 1011
1100100110001000000101011
11101000100000101001000011
11100110000001001001000101
11000101001000000110010101
11000001001100001000101011
100101000101000100000100111
101001100010000000101000111
101010000010010001000011101
110101000000101000010010011
110010010100001000100001011
111000010000010100000110011
110110000000101000100100011
111000000100100010001001101

w=11

1110110111111
11101100000011010111
1101010001000100100 01111
1110100001010010001100101
11100000110001001000011011
11001010010000011010100101
11010010100100010000011011
11100001000010110001000111
110011000001010000100100111
101100010011000000001110011
101011001010000010001001011
101001100100001100001010101
110010010010001010100000111
110100110000010001010010011
110101000100101000001000111
111000100001100001001001101

w=12

11110111011111
1110101001001011001111
10110011010010000000011111
10111001000000110100101011
1110000100110001000101100111
1101000101001000101000011111
1110001000110000011001010111
1011010011000001010101000011
11101010000010011000010011101
11100100010001101000100101011
11000110100010101000000100111
11100100110001000010010010111
11010101001000000110001001011
11100001001000100000110011011
11011000010100100000101010011
11011010000101000100100000111

w=13

111111011101111
111101100000101011011 1
1110001011000010011010011 1
110110010000101110001010011
10110101000100110000101010111
11010110001000011001001001111
11101000101000010010001110111
11100010010011010000110001111
111010100100100000011100010011
101110000110010000100101010111
110101001010000010001100101111
111001000010101001000011010111
110100010001100101000010110111
101001101001010000100010011111
110011010000011000101001001111
110010111000001010100000110111

w=14

1111101111011111
11111100010010101011011
110111010000010100110101011
1110001001110010010010010111
11011100100100000100101010111
10110010100010110010001100111
11100101010000110011000011011
11101010011000010101010000111
101010100011010001100000110111
111001000100110101000010011011
110011001011000100000101101011
110100110000110001001100100111
111100000111000100010100110001 1
110101100010001000010110010111
111010010100100011100000101011
110100100101010010000111001011

w=15

111110110111101111
1111011010001100011011011
110101001010001101100010101111
1110010111000001010001001110111
11101000100011001010100100101111
11100100101100000000111010011011
11011101000100001010001110010111
11101100001001011000011001010111
110110100100010100011001000011111
110101000011010000110100011010111
111001010001011001001000010011111
110100011100100010010101010001111
111010010001100100100010111000111
110110001010101001000011000101111
110011110000100101000100001110111
111001100110000100010010101001111

w=16

1111111011011101111
1101101100001100101010110111
11110001010100010100100011100101
111001001010010000011100000011110111
1101011010000001100010110000100010111
1101010110001001010010010010000011111
1101100010001110001000001100010110111
1011000111000100100010100110001001111
11010101001000000101110000001001011001111
110101000101000110000000101010011010111
1110001110011000000000100011010010101111
111101001010000000110011001001000101010
1101011001001100000000100101010100011111
11011001000100101010001000011010001111
111000110010001001100001000000110011111
111100000100101001010000100110001101111

w=17

11110111011110101111
111001110100011001001001011111
11101001001100000101001110011001111
111001001011001000001110000001011100111
11001110001010001000110100000101001111
11111000000101001100000100010110101111
101101010110000100100001101010001101111
111001101010010010001010010001001101111
101101001011100000000101000111010010011
111001001100110100000101001000101011011
110101010010111000010001000100011010111
111110000100010110001100100110000010111
11100100110000000101101010000001010101111
1110101100100011000100000100001101011111
11010110000100010110001001010000011111
11011001010010000100001011100100100111

w=18

1111101110111110111111
11111101000011001001010111 1011

w=19
